# Detecting Cataract Using Smartphones

**DOI:** 10.1109/JTEHM.2021.3074597

**Published:** 2021-04-20

**Authors:** Behnam Askarian, Peter Ho, Jo Woon Chong

**Affiliations:** 1 Department of Electrical and Computer EngineeringTexas Tech University 6177 Lubbock TX 79409 USA; 2 Lubbock Eye Clinic Lubbock TX 79410 USA

**Keywords:** Cataract, image processing, luminance-based method, smartphone

## Abstract

Objective: Cataract, which is the clouding of the crystalline lens, is the most prevalent eye disease accounting for 51% of all eye diseases in the U.S. Cataract is a progressive disease, and its early detection is critical for preventing blindness. In this paper, an efficient approach to identify cataract disease by adopting luminance features using a smartphone is proposed. Methods: Initially, eye images captured by a smartphone were cropped to extract the lens, and the images were preprocessed to remove irrelevant background and noise by utilizing median filter and watershed transformation. Then, a novel luminance transformation from pixel brightness algorithm was introduced to extract lens image features. The luminance and texture features of different types of cataract disease images could be obtained accurately in this stage. Finally, by adopting support vector machines (SVM) as the classification method, cataract eyes were identified. Results: From all the images that we fed into our system, our method could diagnose diseased eyes with 96.6% accuracy, 93.4% specificity, and 93.75% sensitivity. Conclusion: The proposed method provides an affordable, rapid, easy-to-use, and versatile method for detecting cataracts by using smartphones without the use of bulky and expensive imaging devices. This methodcan be used for bedside telemedicine applications or in remote areas that have medical shortages. Previous smartphone-based cataract detection methods include texture feature analysis with 95 % accuracy, Gray Level Co-occurrence Matrix (GLCM) method with 89% accuracy, red reflex measurement method, and RGB color feature extraction method using cascade classifier with 90% accuracy. The accuracy of cataract detection in these studies is subject to changes in smartphone models and/or environmental conditions. However, our novel luminance-based method copes with different smartphone camera sensors and chroma variations, while operating independently from sensors’ color characteristics and changes in distances and camera angle. Clinical and Translational Impact—This study is an early/pre-clinical research proposing a novel luminance-based method of detecting cataract using smartphones for remote/at-home monitoring and telemedicine application.

## I. Introduction

**Fig. 1.**
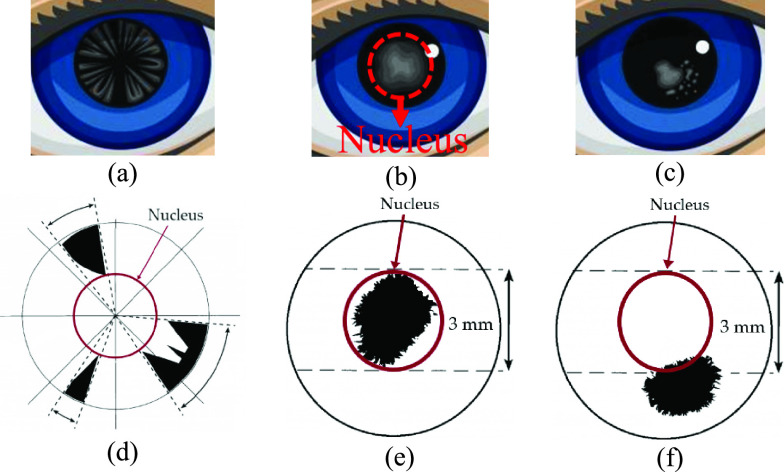
Different types of cataract disease in the eye: (a) cortical cataract, (b) nuclear sclerotic cataract, (c) posterior capsular cataract, (d) demonstration of cortical cataract and its location on the lens, (e) demonstration of nuclear sclerotic cataract and its location on the lens, and (f) demonstration of posterior capsular cataract and its location on the lens.

Cataract is mymargin an eye disease that causes permanent blindness when not treated in time. According to the National Eye Institute, 24.4 million Americans have cataracts, and it is estimated that the number of people affected by cataracts will increase to 38.7 million by 2030 [1]. In 2002, the World Health Organization (WHO) published a simplified cataract grading scale [2]. According to the WHO, there are three main types of cataracts: 1) nuclear sclerotic cataract (NS), 2) cortical spoking cataract (CS), and 3) posterior subcapsular cataract (PSC) which are shown in Figs. 1a, 1b, and 1c, respectively.

Cataract types are defined by the location where the opacities exist within the lens and are graded by how severe the opacities are at that location. Depending on the location of the opacities, as shown in Fig. 1d, if the opacity exists in the outer circle of the lens, causing spoke/wedge-like peripheral cloudiness, it is known as a cortical spoking cataract (CS). If the opacity is in the central portion of the lens (shown in Fig. 1e), it is known as a nuclear sclerotic cataract (NS), which is cloudiness of the nucleus (central optical zone). Finally, if the opacity is in the posterior capsule of the lens (shown in Fig. 1f), it is known as a posterior subcapsular cataract (PSC) [2].

Image processing and machine learning algorithms have been developed to detect eye diseases by analyzing eye image features [3], [4]. In addition, image analyses have been proposed for a computerized ophthalmic diagnostic system that can be used in the diagnosis of some common diseases like diabetic retinopathy, keratoconus, or glaucoma [3]–[9]. Similarly, smartphone applications have been used for remote health monitoring such as remote eye care [5], [8], [10]. Smartphones have also been used to detect cataracts by using lens color and texture features [11]–[15].

Fuadah *et al.* have used a combination of statistical texture features and adopted the K-Nearest Neighbor (k-NN) algorithm as a classifier to detect cataracts on an Android-based smartphone [11]. In their study, they have utilized the Gray Level Co-occurrence Matrix (GLCM) to distinguish cataracts from healthy eyes. GLCM can extract texture features and measure contrast variations. The accuracy of their method utilizing the k-NN classifier was 95%. Kaur *et al.* have utilized an external microscope on an Android-based smartphone to detect cataracts from Red, Green, Blue (RGB) images captured from the subject’s retina. The reported accuracy of their method was 89% [13]. Moreover, Lau *et al.* proposed a self-screening application for detecting cataracts based on red reflex measurements [14]. Their system replicates red reflex generated by ophthalmoscope using a smartphone flashlight. Their system uses Artificial Neural Networks (ANNs) to distinguish healthy eyes from cataracts. The system was implemented on a Xiaomi Mi3 smartphone. Furthermore, Rana *et al.* adopted a cascade classifier to detect the pupil and extract RGB feature values [15]. After feature extraction, they used OpenCV functions to compare the pupil color with the database and determine whether the subject has cataracts or not. They examined 50 subjects, 20 with cataracts and 30 healthy subjects. The accuracy of detecting cataracts using their method was 90%.

Since these studies are highly dependent on smartphone camera sensor characteristics as well as ambient light, distance, and environmental conditions, the accuracy of detecting cataracts in these studies is subject to changes in smartphone models and/or environmental conditions.

To overcome these disadvantages of color-based methods used in the mentioned studies, we propose an alternative luminance-based method for cataract disease diagnosis. Luminance-based models have been used in different studies to detect diseases such as cancer and have been compared with color-based detection methods in different color spaces [16]–[24].

In [22], the hue, saturation, value (HSV) color-based method and a luminance-based method were compared to detect cancer cells in leukemia. The study concluded that the luminance-based method had 86.67 % accuracy while the HSV color-based method had only 33.4% accuracy detecting cancer cells. In [23], the authors compared the RGB color-based method with the luminance-based method to detect white blood cells in myeloid leukemia. The results of their study showed that implementing luminance-based method improves the accuracy of detecting the disease by 23% compared to the RGB color-based method. In [24], the authors compared the RGB color based method with the luminance-based method for detecting prostate cancer. Their study concluded that changing the method from RGB color-based to luminance-based increases the detection accuracy by 13.2%. In [25], the authors proposed a multi-feature prostate cancer diagnosis technique using luminance-based method and compared their method with RGB color-based method. The results of their study showed that adopting luminance-based method increases the accuracy of detection by 17% compared to the RGB color-based method.

The results of these studies indicate that switching from color-based method to luminance-based method improves the detection accuracy and feature extraction. Moreover, color-based detection systems are more dependent on the camera sensors’ characteristics and environmental conditions compared to luminance-based detection methods.

In this paper, we propose a novel and robust luminance-based eye image analysis technique. Specifically, we detect cataracts by using pixel brightness transformation to extract luminance values of images acquired with a smartphone camera. To validate the method, we used different iPhone models (iPhone 6, iPhone X, and iPhone 11 Pro) to capture images in different light, distance, and angle conditions using an eye model [26]. The luminance-based images were then cropped and transformed using the watershed transformation algorithm to extract the region of interest (ROI).

The novel luminance-based method copes with different smartphone camera sensors and chroma variations, and it is independent of sensors’ color characteristics and changes in environmental factors. For classification, the Support Vector Machine (SVM) classifier was utilized to distinguish diseased eyes from healthy eyes. To overcome the data sample limitation, a 10-fold cross validation resampling procedure was implemented to evaluate the machine learning model. As a result, the proposed method detects cataracts from images captured by the smartphone camera. The proposed method only differentiates healthy eyes from diseased eyes, and it does not differentiate between types of cataracts.

The rest of this paper is organized as follows: Section 2 describes the data acquisition, preprocessing, luminance transformation, and classification methods. Section 3 explains the results of the classification and presents the image features. Finally, Section 4 concludes the paper.

## II. Methods and Procedures

**Fig. 2.**
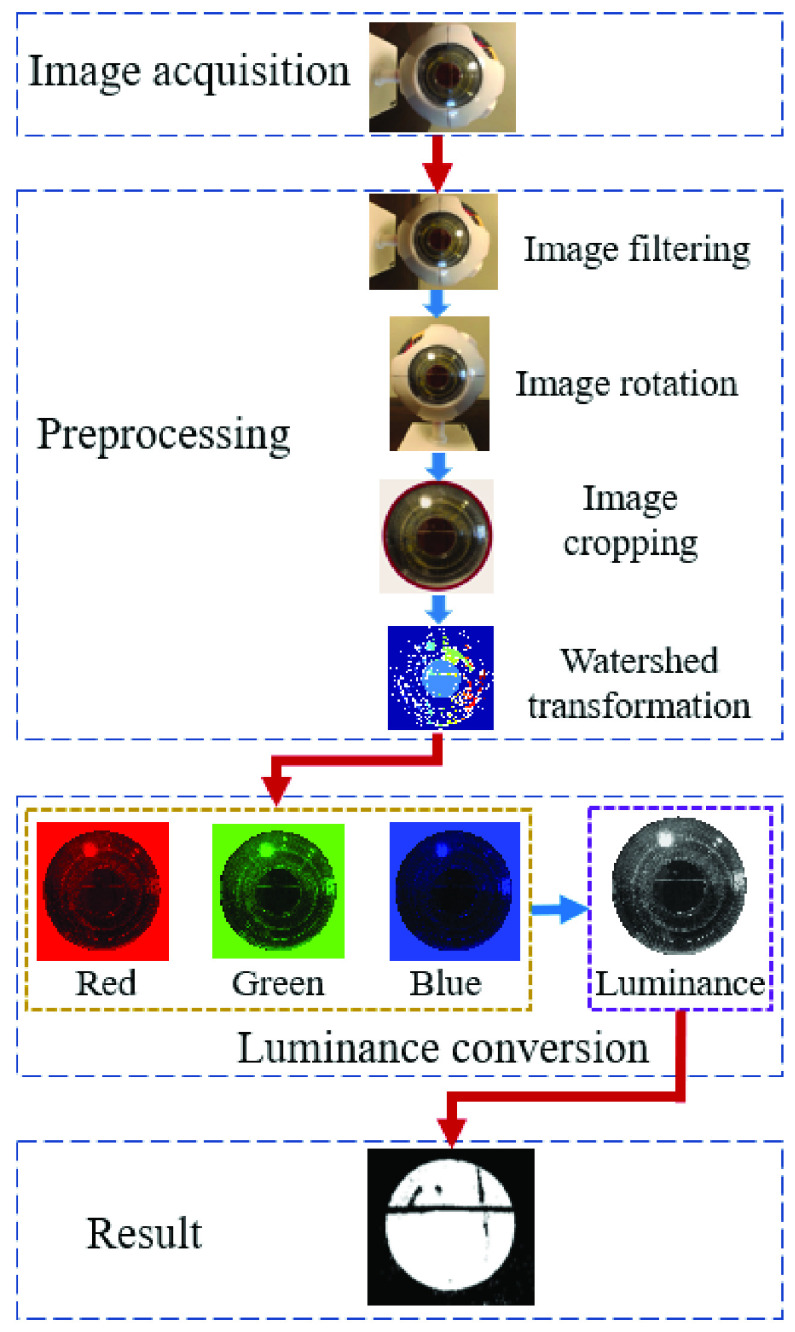
Flowchart for detecting cataracts using the luminance-based method. The method starts with image acquisition using a smartphone camera. In the preprocessing step, the acquired image is filtered for noise removal, occasionally rotated, and cropped to extract the region of interest; then watershed transformation is applied for segmentation. In the next step, the R, G, and B components are converted to luminance, and finally, the image is classified to extract cataract features. The red arrows are the transitions between the main steps (image acquisition, preprocessing, luminance conversion, and result), and the blue arrows, show the transitions between sub steps (e.g., image filtering to image rotation, etc.).

Images captured by the smartphone camera are utilized to monitor the conditions of the eye for healthy or cataract cases. Our proposed method to diagnose cataracts consists of four main steps: 1) data acquisition, 2) preprocessing, 3) feature extraction, and 4) classification. Fig. 2 shows the processing flowchart for detecting cataracts.

### A. Data Acquisition

In this paper, we used an Axis Scientific 7-Part Human Eye ($5\times $ Life Size) eye model to emulate healthy and diseased eyes in different environmental conditions [26]. Images from eye models were acquired using iPhone X, iPhone 6, and iPhone 11 Pro smartphone cameras. The camera settings used were the LED flashlight, autofocus, and maximum resolution.

**Fig. 3.**
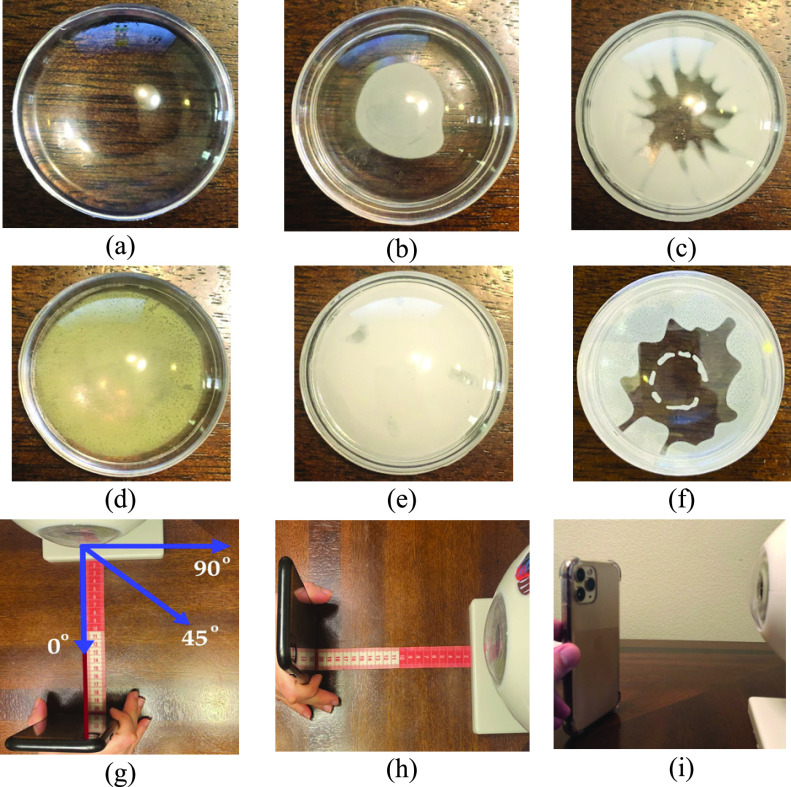
Demonstration of different type emulations on the model eye lens: (a) healthy lens, (b) posterior subcapsular cataract, (c) cortical cataract, (d) nuclear cataract, (e) mature cataract, (f) capsular cataract, (g) demonstration of camera angles, (h) distance measurement procedure, and (i) adoption of different smartphones for data acquisition (iPhone 11 Pro).

In the experiment setup, the subjects will sit in a relaxed position while keeping their head and eye in a stable position and align their eye with the smartphone’s rear camera. The camera can be located between 10 cm to 50 cm distance from the eye with autofocus to have a clear image from the eye. After images are captured, the smartphone will process the images and present the results. For this paper, 100 eye model images were captured, 50 from healthy eye models, and 50 from diseased eye models. The eye model was equipped with different lenses emulating the diseased eye with different types of cataracts, including a posterior subcapsular cataract, a cortical cataract, a nuclear cataract, a capsular cataract, and a mature cataract. The disease emulation on the model eye is shown in Fig. 3. Fig. 3a shows the healthy eye model and Figs 3b, 3c, 3d, 3e, and 3f show the model eye with posterior subcapsular cataract (opacity is in the posterior capsule of the lens), cortical cataract (opacity exists in the outer circle of the lens, causing spoke/wedge-like peripheral cloudiness), nuclear cataract (cloudiness of the nucleus -central optical zone), mature cataract (the lens is totally opaque), and capsular cataract (opacity is in outer layer of the lens), respectively. According to the National Eye Institute (NEI), all of these types of cataracts are considered to be diseased eyes [27].

We diversified the environmental factors to evaluate the impact of each factor on image features and the results. Specifically, we selected four main environmental factors that would impact the results: 1) ambient lighting, 2) distance between the smartphone camera and eye model, 3) camera angle relative to eye model, and 4) smartphone camera characteristics. Demonstration of different distances, angles, and smartphones are shown in Figs 3g, 3h, and 3i, respectively. The 50 images acquired from healthy eye models comprised of 10 images from 10 cm distance, 10 images from 20 cm distance, 10 images from 30 cm distance, 10 images from 40 cm distance, and 10 images from 50 cm distance by varying the smartphones and camera angles. For the diseased dataset, we acquired 50 images from 5 types of the cataract eye models which comprised of 10 images of posterior subcapsular cataracts, 10 images of nuclear cataracts, 10 images of cortical cataracts, 10 images of mature cataracts, and 10 images of capsular cataracts using different distances, smartphones, and camera angles.

To investigate the effect of each environmental factor on image features, we evaluated the effect of that factor on image features by constraining all other environmental factors and changing only one factor at each data acquisition stage. Hence, we had four phases of validation for environmental factors as follows:

• Fixed distance, camera angle, and smartphone to 20 cm, 0-degree, and iPhone X, respectively, while increasing ambient light intensity from 1600 lumens to 6100 lumens with step size of 1500 lumens.
• Fixed ambient light intensity, camera angle, and smartphone to 3100 lumens, 0-degree, and iPhone X, respectively, while increasing the distance from 10 cm to 50 cm with step size of 10 cm.
• Fixed distance, ambient light intensity, and smartphone to 20 cm, 3100 lumens, and iPhone X, respectively, while changing camera angle directions into 45° upside, 45° downside, 45° left side, 45° right side, and 0° center.
• Fixed distance, camera angle, and ambient light intensity to 20 cm, 0-degree, and 3100 lumens, respectively, while changing smartphone to iPhone 6, iPhone X, and iPhone 11 Pro.

The different ambient light environments were set up in a dark room where the light sources were picked using dimmable lights and the color Muse device [28] was utilized to measure environmental light continuously in a precise way.

### B. Luminance Calibration

**Fig. 4.**
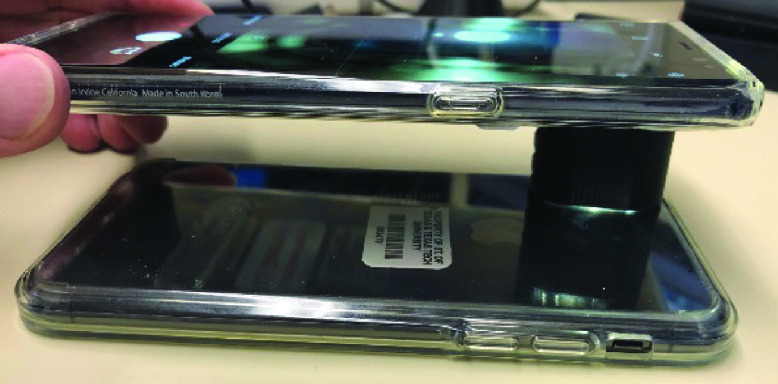
Luminance calibration of the smartphone camera sensor. Two identical iPhone X phones are used for calibration. One smartphone is used as a light source and the other one as the light sensor. The process was repeated the other way around.

Since we used 3 different smartphones in this study, each having their own camera sensor characteristics and flashlight specifications, we had to calibrate the camera sensor and measure the amount of light coming from the smartphones’ flashlights. To calibrate the luminance reflection from different smartphones, we first measured the maximum luminance coming from an identical smartphone’s flashlight as shown in Fig. 4 and used that as the gold standard. To eliminate ambient light and minimize the loss of luminance during the calibration process between identical smartphones, we designed and manufactured a special gadget using a 3-D printer from Acrylonitrile Butadiene Styrene (ABS) [29], which is a dark absorbance material. The 3D-printed gadget is an elliptical cylinder with a minor axis of 13 mm, major axis of 25 mm, and a height of 15 mm as shown in Fig. 4.

Next, we switched to different smartphones to calibrate the flashlight power. The flashlight power on each phone is adjustable and we tuned the flashlight to have a controlled and fixed amount of emitted light from each smartphone’s flashlight. Specifically, the emitted light from iPhone 6, iPhone X, and iPhone 11 Pro’s flashlights were measured at 112, 136, and 154 lumens, respectively, and we adjusted all smartphone flashlight intensities to 100 lumens using the smartphone’s flashlight tuning application [27]–[29].

### C. Preprocessing

**Fig. 5.**
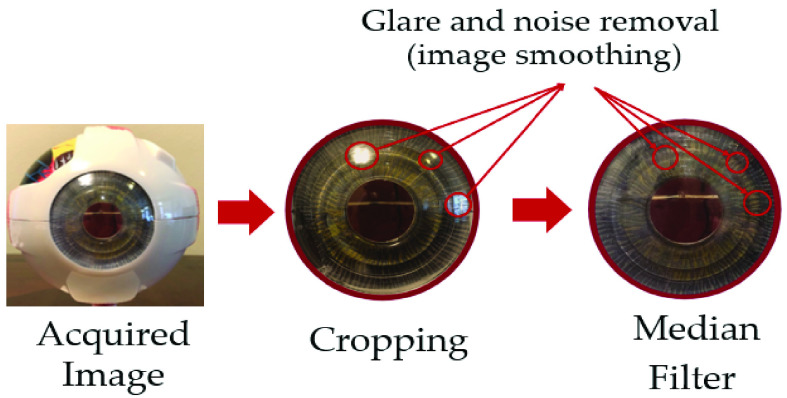
Median filter and preprocessing demonstration: Images acquired from the smartphone are cropped using circle cropping to extract the ROI. Next, the cropped image is filtered using the median filter to extract glare and noise and smooth the image.

The preprocessing step is necessary for effective and accurate feature extraction of the images. The two main parts of the preprocessing step are image segmentation and denoising. Images captured by a smartphone camera include other parts of the eye (e.g., iris, conjunctiva). Hence, image segmentation is required to extract the cornea region from the original image. The image segmentation was performed by using semi-automatic cropping of the eye image to extract the region of interest (ROI). The cropping was performed using a circle mask by marking the center of the circle and the radius of the circle. Here, the cornea was extracted from the eye image as shown in Fig. 5.

Taking into consideration that noise has a negative impact on acquired images, it is necessary to denoise the images using median filtering before processing to reduce the impact on eye segmentation and identification caused by irrelevant background in the images. The median filter has been widely applied in removing pepper salt and glare noise from an image while preserving edges and keeping main color feature information. In this study, the median filter was utilized to preprocess and smoothen the source images as shown in Fig. 5.

An example of pepper salt denoising is shown in Fig. 5, in which we marked some examples of noise using red circles to visualize how the procedure is done and what the restored image looks like [30]. The ROI in the eye model is the cornea part of the eye, which is cropped out in the first step. The median filter is then applied to eliminate noise and glare as shown in Fig. 5. In addition, the watershed segmentation algorithm was adopted to extract the lens boundaries and to extract the ROI from the filtered image.

### D. Watershed Transform

**Fig. 6.**
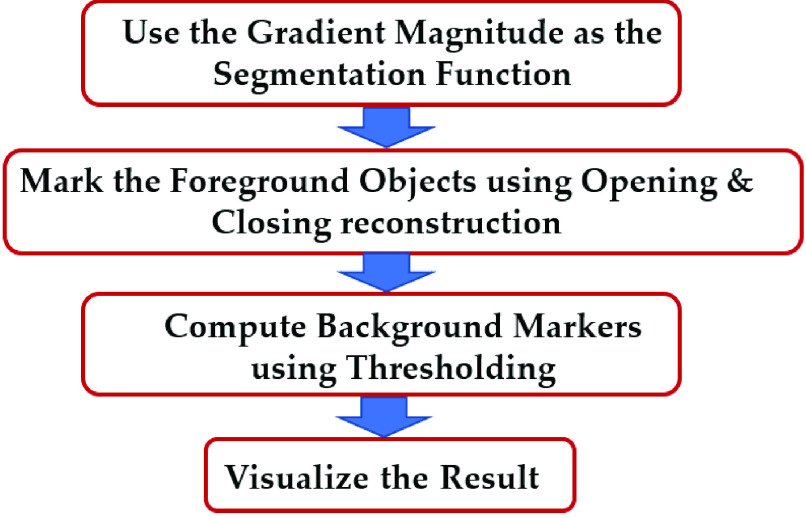
Watershed algorithm flowchart.

**Fig. 7.**
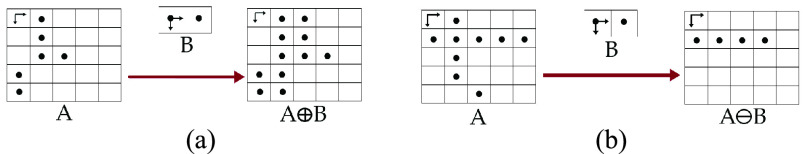
Illustration of dilation and erosion morphological operations: (a) example of dilation using $1\times 2$ structural element B on the sample image A, and (b) erosion operator example using a $1\times 2$ structural element B on the sample image A.

Because most cataract diseases appear in the shape of a circle in the lens area, the watershed algorithm can be applied for image segmentation and visualization of the diseased areas [31], [32]. The watershed algorithm was performed in 4 steps, as shown in Fig. 6, starting with running a gradient operator for edge detection. The next step after gradient magnitude extraction was opening and closing reconstruction operators. Opening removes small objects, while closing removes small holes. After the opening-closing reconstruction step, the thresholding algorithm was adopted to eliminate dark spots in the background. Finally, we visualize the results of the watershed algorithm. The following flowchart shows the mentioned steps of the watershed algorithm:

Gradian magnitude is calculated using the following equation [33]:

\begin{align*} \nabla f=\left [{ {\begin{array}{cccccccccccccccccccc} g_{x}\\ g_{y}\\ \end{array}} }\right]=\left [{ {\begin{array}{cccccccccccccccccccc} \frac {\partial f}{\partial x}\\[0.3pc] \frac {\partial f}{\partial y}\\ \end{array}} }\right],\tag{1}\end{align*}

where $\frac {\partial f}{\partial x}$ is the derivative with respect to x (gradient in the x-direction), and $\frac {df}{dy}$ is the derivative with respect to y (gradient in the y-direction). Opening reconstruction is the dilation of the erosion of a set A by a structuring element B, and the equation is as follows [34]:

\begin{equation*} A \circ B=\left ({A \ominus B }\right)\oplus B,\tag{2}\end{equation*}

where “$\circ $” is the opening operation, $\oplus $ denotes dilation, and $\ominus $ denotes erosion. Dilation and erosion are two fundamental morphological operations. Dilation expands image pixels by adding pixels to the object boundaries of an image, while erosion compresses image pixels by removing pixels on object boundaries. Dilation in a binary image is defined by [34]:

\begin{equation*} A\oplus B=\bigcup \nolimits _{b\in B} A_{b},\tag{3}\end{equation*}

where $\cup $ is union operator and $A_{b}$ is the translation of $A$ by $b$. Erosion is defined by the following expression [34]:

\begin{equation*} A\ominus B=\bigcap \nolimits _{b\in B} A_{-b},\tag{4}\end{equation*}

where $\cap $ is intersection operator and $A_{-b}$ denotes the translation of $A$ by $-b$. The dilation and erosion operators on a sample image are shown in Fig. 7. In this figure a $1\times 2$ structural element $B$ is used were all $b = 1$ to execute the erosion and dilation operations.

We implement the closing reconstruction algorithm which is the erosion of dilation of image A by a structuring element B, using the following equation [34]:

\begin{equation*} A\bullet B=\left ({A\oplus B }\right)\ominus B,\tag{5}\end{equation*}

where $\bullet $ is the closing operation.

**Fig. 8.**
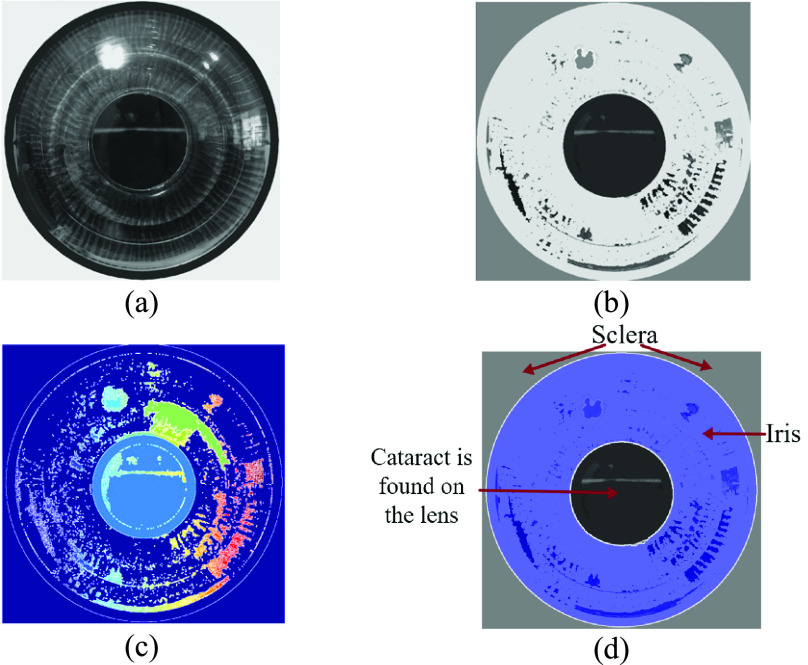
Results of watershed algorithm: (a) luminance based transformed image, (b) gradient magnitude, (c) foreground objects marked using opening ($A\circ B$) and closing ($A\bullet B$) reconstruction, this figure is the result of Eqns. 2, 3, 4 and 5, and (d) background marks computed, and regional maxima superimposed on the original image, visualizing the result.

Fig. 8 shows the steps of the watershed segmentation algorithm on a sample eye image. First, the images are generated using the luminance transformation method (Fig. 8a). This figure is used to calculate the gradient magnitude which is shown in Fig. 8b to detect edges and boundaries. Next, the closing-opening reconstruction and watershed transform is implemented on the gradient magnitude to segment different regions (Fig. 8c). Finally, the segmented areas are superimposed on the original luminance-based image using the watershed algorithm as regional maxima as shown in Fig. 8d.

### E. Feature Extraction

**Fig. 9.**
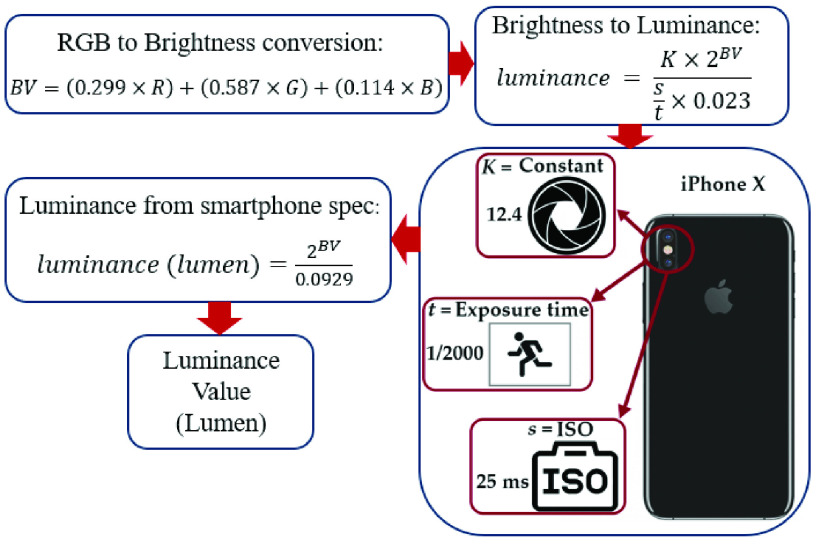
Flowchart of luminance extraction from preprocessed RGB image: The preprocessed RGB image is transformed to extract the brightness values. The brightness values are then converted to pixel luminance values using smartphone camera’s optical specification.

Our proposed cataract detection method uses luminance values reflected from an eye to detect cataracts. In a smartphone, the luminance value can be determined by exposure time ($t$), ISO speed ($s$), light meter constant ($K$), and brightness values (*BV*) [35]. These values are fixed, although they depend on the smartphone model specifications, and these specifications vary depending on which smartphone model is used for image acquisition. However, the brightness values (*BV*) change depending on the acquired images [29]. For the iPhone X smartphone, which is used in this study, the camera’s $K$, $s$, and $t$ are set to 12.4, 25 ms, and 1/2000 s, respectively. To measure luminance value from an image captured by a smartphone camera, the proposed algorithm calculates brightness values (*BV*) from the *R, G,* $B$ values of the image. Next, by using the smartphone camera’s specifications, the proposed algorithm converts the brightness into luminance values. Fig. 9 shows the flowchart of extracting luminance values of each pixel from its *R, G,* $B$ values. As shown in Fig. 9, we first need to extract brightness values from measured *R, G,* $B$ values to measure luminance values using the smartphone’s camera. Brightness values (*BV*) are calculated using the following equation from *R, G,* $B$ channel values [36]:

\begin{equation*} BV=\left ({0.299\times R }\right)+\left ({0.587\times G }\right)+\left ({0.114\times B }\right),\tag{6}\end{equation*}

where $R$, *G,* $B$ are the red, green, and blue channel values of each pixel, respectively. Using the above equation, we determined the brightness value for each pixel of the eye image. Next by adopting the following equation, we convert brightness values to luminance values [36], [37].

\begin{equation*} luminance\left ({lumen }\right)=\frac {K\times 2^{BV}}{\frac {s}{t}\times 0.023},\tag{7}\end{equation*}

By replacing the $K$, $s$, and $t$ values in Eq (7) with the values from iPhone X, we reach the following equation which is the equation we used to extract luminance values of each pixel in our method [37].

\begin{equation*} luminance\left ({lumen }\right)=\frac {12.4\times 2^{BV}}{\frac {25\times {10}^{-3}}{\frac {1}{2000}}\times 0.023}=\frac {2^{BV}}{0.0929},\tag{8}\end{equation*}

To evaluate the performance of the luminance-based method, we compared our method with a generally used color-based method utilizing RGB color space. The comparison was performed on 15 randomly picked images out of 50 images from the diseased subset. Specifically, the RGB color-based method utilized by Rana *et al.* was implemented, and the results were compared with our luminance-based method [15]. In their method, they used the cascade classifier to extract the pupil and RGB temporal matrix values to detect cataracts. Table 1 shows mean and standard deviation (SD) values of RGB and luminance from the images of the emulated eye with nuclear cataracts.

**TABLE 1:** Mean and Standard Deviation Values for Luminance and RGB Channels From Emulated Eyes With Cataract Disease

Channel	Red (R)	Green (G)	Blue (B)	Luminance
Mean ± SD	167.5 ± 17.8	71.3 ± 18.36	128.2 ± 24.9	140.7 ± 16.7

**Fig. 10.**
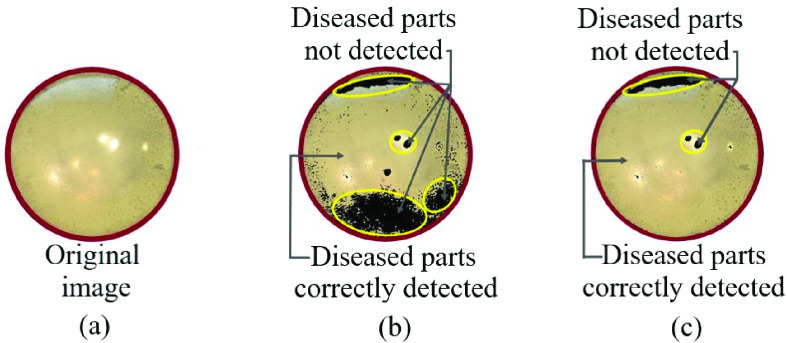
Comparison between luminance based and RGB color-based method: (a) Original eye model image with nuclear cataracts, (b) result of RGB color-based detection method, and (c) result of luminance-based cataract detection method.

Fig. 10 shows the comparison between the luminance-based method and color-based method on a sample emulated eye with nuclear cataracts. Fig. 10a shows the original image from the eye model with a nuclear cataract, Fig. 10b shows the result of the RGB color-based detection method and Fig. 10c shows the result of luminance-based cataract detection method. The cataract detected parts are shown in transparent color and the healthy parts are shown with dark color. The misclassified parts are marked with yellow color, and as shown in Figs. 10b and 10c, the misclassified parts in the color-based method are significantly larger. After applying both methods on the 15 nuclear cataract images, we realized that the luminance-based method has a noticeable advantage over the color-based method, as it was able to detect 36% more diseased pixels compared to the color-based method (shown in Fig. 10).

### F. Classification

In this step, we used the mean luminance values of lens images for classification and differentiation of cataract images from healthy images. The images from both classes are randomly divided into the following subsets: training, validation, and testing. 70% of the data (70 images) were allocated for training and validation, and the remaining 30% (30 images) for testing. Moreover, we applied the support vector machine (SVM) for the classification step. The SVM can be efficiently implemented on smartphones, and it has been adopted for eye disease diagnosis [38], [39].

In this paper, we adopt Gaussian kernel for the SVM classifier to distinguish our samples into healthy and diseased classes. To prevent overfitting, 10-fold cross-validation was performed on the training and validation data. Therefore, 9-folds were used to train the SVM model and 1-fold to validate the model.

## III. Results

**Fig. 11.**
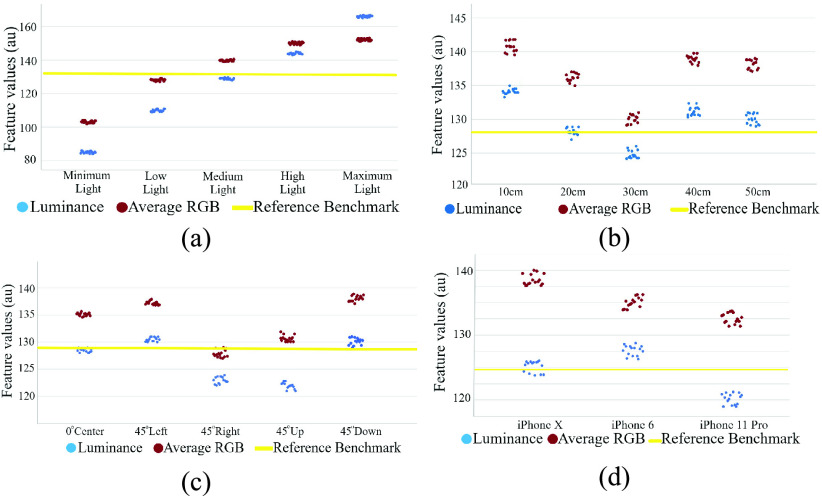
Demonstration of different environmental effects on the luminance and RGB color features: (a) average RGB and luminance values in different ambient light conditions, (b) result of distance variation on luminance and average RGB values, (c) luminance and average values of different angles, (d) luminance and RGB values of the different smartphones. The reference benchmark (represented with yellow line) measurement was acquired using iPhone X from 20 cm distance at medium light conditions.

Images from 100 eye samples of different environments were gathered in this paper. Among these 100 samples, 50 were diseased eye samples and the other 50 were healthy samples. First, we evaluated the impact of different environmental factors on image features using 15 randomly picked images, among 50 images for each environment, by constraining three factors and altering the fourth factor. We repeated this by alternating all four environmental factors one by one. The results of the impacts of different environmental factors on RGB and luminance values are shown in Fig. 11. In Fig. 11 the luminance values are shown with blue dots, the average RGB values are shown with red dots, and the benchmark is marked with a yellow line. The impacts of the different light environments are shown in Fig. 11a. Here, the benchmark (yellow line) is the state in which we fixed each environmental factor to gather the images. The benchmark images were acquired at 20 cm distance, from a medium light environment, from the center angle, using the iPhone X. To evaluate the effects of different environmental factors on image features, we first calculated the mean and SD of the luminance value and average RGB value at the minimum and maximum status in each graph. Then we calculated the differences between the means of the maximum and minimum values to calculate the effects of the environmental factors on RGB and luminance values. The percentage difference was calculated using the following equation:

\begin{equation*} Difference\% =\frac {\left |{ X_{Max}-X_{Min} }\right |}{\frac {X_{Max}+X_{Min}}{2}}\times 100\%\tag{9}\end{equation*}

where $X_{Max}$ is the average maximum luminance or RGB value, and $X_{Min}$ is the average minimum RGB or luminance value.

The results of changing ambient light while fixing other environmental factors is shown in Fig. 11a. The results of changing the distance while fixing other environmental factors is shown in Fig 11b. The results of changing the angle while fixing other environmental factors is shown in Fig 11c. Finally, the results of changing the smartphone while fixing other environmental factors is shown in Fig 11d.

**Fig. 12.**
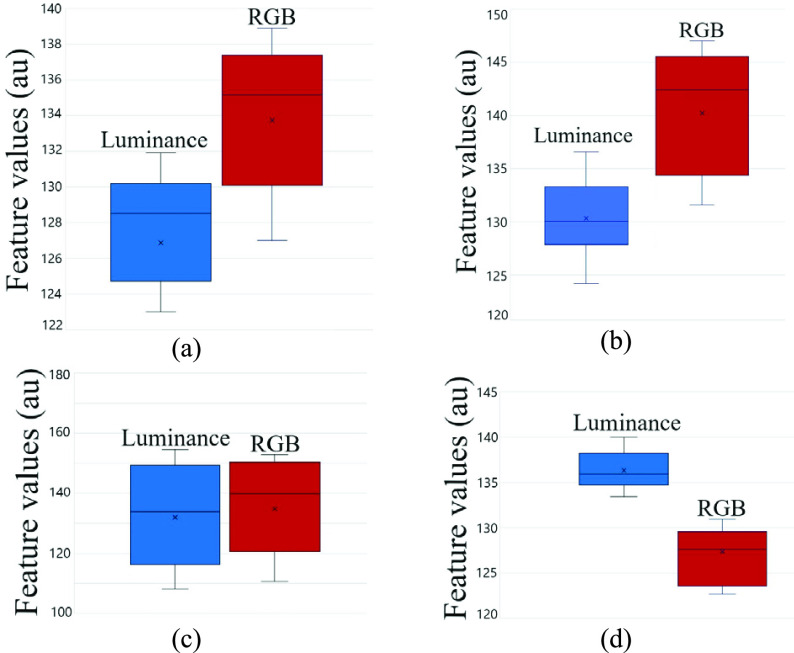
Differences between luminance-based method and color-based method in different environments demonstrated with boxplot graph: (a) difference between luminance-based and RGB color-based method in different angles, (b) difference between luminance-based and RGB color-based method in different distances, (c) difference between luminance-based and RGB color-based method in different ambient lights, and (d) difference between luminance-based and RGB color-based method in different smartphones.

Fig. 12 shows the effects of different environmental factors on RGB and luminance values using box plots.

As shown in Figs. 12a, 12b, 12c, and 12d, and by using Eq. 9, we noted that changing the camera angle, distance, and smartphone had 2.2%, 3.3%, and 3% impact on luminance values and 9.2%, 13.3%, and 8.5% impact on RGB values, respectively. On the other hand, changing the ambient light had 36% difference impact on the luminance values, which was similar to the 32% difference impact it had on the RGB values. Hence, Fig. 12 shows that changing environmental factors has less effect on luminance values compared to RGB values. To determine the difference between healthy and diseased luminance values, we conducted the statistical hypothesis test to find the $p$-values. The paired-samples $t$-test was conducted to compare the luminance values and determine if there is a significant difference between the healthy and diseased luminance values. The $p$-value from the paired $t$-test was equal to $3.3\times {10}^{-7}$ which is less than $p < 0.05$ and shows a significant difference between the luminance values of a healthy and diseased eye. The results revealed that there was a significant difference between healthy (Mean = 94.7, SD = 4.1) and diseased (Mean = 140.2, SD = 19.4) luminance values ($p < 0.05$).

**Fig. 13.**
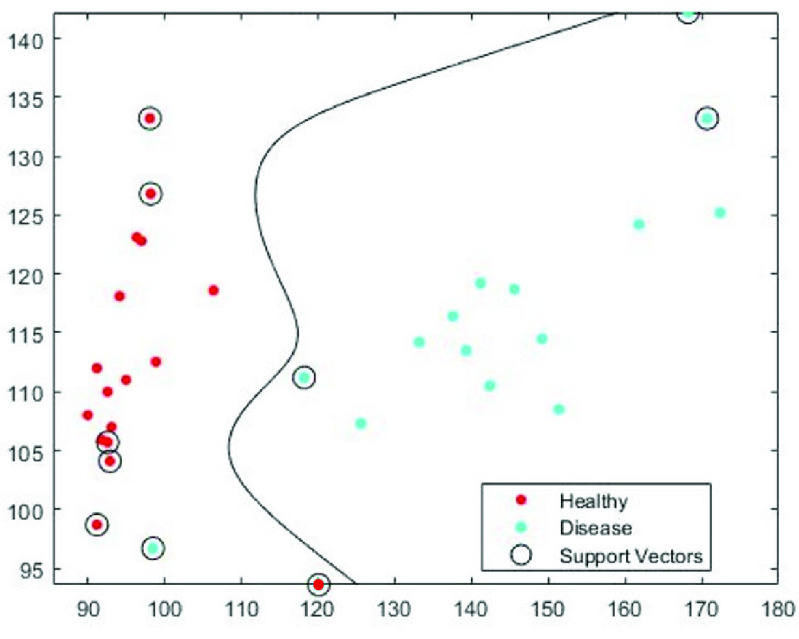
Support Vector Machine (SVM) classifier diagram with Gaussian kernel.

Fig. 13 shows the results of the SVM classifier on color and luminance features on a testing subset of 15 healthy and 15 diseased images. These images were randomly picked among 50 healthy and 50 diseased images, acquired from the model eye. The testing subset (15 healthy + 15 diseased) were randomly selected and left out during the training session. In Fig. 13, the SVM classifier with Gaussian kernel was used to distinguish healthy and diseased classes using the luminance values and red channel values. The support vectors are marked with a circle, the healthy class with red and the diseased class with blue. In all mentioned figures, luminance values are on the x-axis and R color values are on the y-axis.

**Fig. 14.**
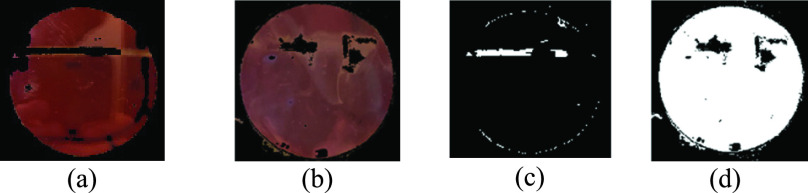
Results of the proposed method: (a) original image of a healthy eye, (b) original image of an eye with cataract, (c) result of cataract detection on the healthy eye, and (d) result of cataract detection on the diseased eye.

Fig. 14 shows the results of our proposed method using the luminance-based method. An image from a healthy eye model is shown in Fig. 14a, and an image from a sample diseased eye is shown in Fig. 14b. The result of our proposed method on the healthy eye from Fig. 14a is shown in Fig. 14c, and the results of the method on the diseased eye from Fig. 14b is shown in Fig. 14d. Fig. 14 demonstrates that our proposed method can accurately distinguish cataract features in a diseased eye from a healthy eye.

The performance of our proposed method is evaluated in terms of sensitivity, specificity, and accuracy, which are defined as:

\begin{align*} Sensitivity=&\frac {TP}{TP+FN}\mathrm {\times 100\% },\tag{10}\\ Specificity=&\frac {TN}{TN+FN}\mathrm {\times 100\% },\tag{11}\\ Accuracy=&\frac {TP+TN}{TP+TN+FP+FN}\mathrm {\times 100\%},\tag{12}\end{align*}

where *TP* is true positive (diseased eye correctly diagnosed as diseased), *FP* is false positive (healthy eye incorrectly identified as diseased), *TN* is true negative (healthy eye correctly identified as healthy), and *FN* is false negative (diseased eye incorrectly identified as healthy). Adopting the above equations, the accuracy of detecting the diseased eye with the proposed method was 96.6%, the sensitivity was 93.75%, and the specificity was 93.4%.

Table 2 shows the accuracy performance of the proposed algorithm with 70 training images (35 cataract and 35 healthy images) when the 10-fold cross validation is applied. The average and SD values of accuracies is 98 ± 0.014% as shown in Table 1. We applied the decision boundary obtained from this 10-fold cross validation into the test data set (15 cataracts and 15 healthy images). As a result, we obtained 96.6% accuracy, 93.75% sensitivity, and 93.4% specificity as shown in Table 2.

**TABLE 2:** The Average Accuracy of the 10-Fold Cross Validation and the Specificity, Sensitivity and Accuracy of Testing Dataset

Test data set Specificity	Test data set Sensitivity	Test data set Accuracy	Average 10-fold cross validated accuracy ±STD
0.934	0.9375	0.966	0.98 ± 0.014

**TABLE 3:** Comparison With Other Methods

Research	Accuracy	Limitation
Our method	96.6 %	Works on any smartphone and environment
Fuada et al.	95%	Works on only one smartphone from 20 cm distance, and controlled ambient light
Lau et al.	–	Requires pupil dilation and retinal exam (fundus lens) which is inconvenient.
Rana et al.	90%	Tested on only one smartphone with specific camera sensor characteristics.
Kaur et al.	89%	Works only in 20 cm distance and tested only on Xiaomi M3 smartphone

## IV. Conclusion

In this paper, we have investigated the feasibility of detecting cataracts using smartphones. We have proposed an accurate, portable telemedicine solution which can detect cataracts by adopting a novel luminance-based feature extraction algorithm. This method can be used for bedside, clinical, and community applications, which are the main pillars of translational medicine [40]–[42]. We have implemented our method using different smartphone camera pictures from 100 eye models, of which 50 were cataracts and 50 were healthy. Images were acquired from the eye models emulated to replicate different types of cataract disease, and healthy eyes. Our proposed method aimed to find cloudiness and blurriness as signs of cataracts in the eye model. To evaluate environmental factors, the proposed algorithm was evaluated in different environments to assess the effects of distance, ambient light, angle, and different smartphone camera characteristics on the research outcome. A novel luminance-based method is proposed by extracting luminance features from the brightness and RGB color components. To improve the performance of our proposed method, a median filter was adopted for preprocessing, and a watershed algorithm was used to enhance luminance features and extract noise and glare. To distinguish healthy from diseased eyes, 10-fold cross-validation and the SVM classifier were adopted for the classification task.

Previous methods of detecting cataracts using smartphones include the GLCM method proposed by Fuadeh *et al.* [11] the retinal exam method proposed by Lau *et al.* and Kaur et al, [13], [14] and the RGB color-based texture extraction method proposed by Rana and Galib [15].

The drawback of the GLCM method proposed by Fuadeh *et al.* [11] is that the method was tested only on one smartphone in a controlled environment, and the impact of different smartphone specifications and environments was not studied in their research.

The drawback of the method proposed by Lau and Chan [14] is that the patient’s pupil needs to be dilated for maximum light entrance, which is inconvenient for the patients. The retinal exam captures the image from retina using a condensing lens which must be at a certain distance from the eye. The condensing lens must be at exactly 20 cm distance from the smartphone and 5 cm distance from the eye. However, our method gathers images directly from the lens and does not require the condensing lens, therefore it does not have the issue of certain distance since the image can be acquired from any distance (from 10 cm to 50 cm). In the study conducted by Rana and Galib [15], the drawback is that their system has limitations detecting cataracts when changing the smartphones and the camera color characteristics.

A comparison in terms of accuracy between our method and the mentioned studies has been presented in the following table.

As shown in Fig. 13, even the smallest reflection and light entering between the gaps of the eye model coming from the back of the eye model is distinguished and marked as components of a cataract. Here this extra light is marked as sign of cataracts because the luminance features of these pixels are in the diseased class of the trained classifier. This shows that any anomaly or light reflection is precisely measured using our method. Hence, even the mildest cataracts or lens anomalies can be accurately detected using our proposed method. This shows that our method can detect the pixels (parts of lens) which are affected with cataract disease and it is capable of determining the progress of the disease. The proposed method is also capable of distinguishing between different types of the disease, and it can determine the shape and which parts of the lens are affected (e.g., posterior, nuclear, capsular).

All the mentioned studies have used a single smartphone for image acquisition and acquired the data in a controlled environment. The results of their studies are heavily dependent on the smartphone’s camera configuration and sensor characteristics, the distance between the smartphone and eye, and the camera angle. However, our proposed method is effective irrespective of those environmental factors.

The accuracy, specificity, and sensitivity of the proposed method to detect signs of cataracts was 96.6%, 93.4%, and 93.75%, respectively. Experimental results in this study show that the method can accurately detect cataracts from different distances, smartphones, and camera angles. It also shows that changing environmental factors had a very limited impact (average 2.8% impact) on the outcome results. Alternating the camera distance, smartphone, and camera angle had 3.3%, 3%, and 2.2% impact, respectively on the outcome results, which was negligible. We can implement our method on any smartphone, including iOS or Android phones, with adequate hardware using a re-targetable application platform [43].
